# Long term follow-up of inguinal endometriosis

**DOI:** 10.1186/s12905-021-01235-2

**Published:** 2021-03-02

**Authors:** BoRan Mu, ZhiQiang Zhang, Chongdong Liu, Kunning Zhang, ShuHong Li, JinHua Leng, MengHui Li

**Affiliations:** 1 Department of Obstetrics and Gynecology, Beijing Chao-Yang Hospital Affiliated To Capital Medical University, 8 Gongtinanlu, ChaoYang District, Beijing, 100020 People’s Republic of China; 2 Department of Pathology, Beijing Chao-Yang Hospital Affiliated to Capital Medical University, 8 Gongtinanlu, ChaoYang District, Beijing, 100020 People’s Republic of China; 3 Department of Obstetrics and Gynecology, Peking Union Medical College (PUMC) Hospital, No. 1 Shuaifuyuan Wangfujing, Dongcheng District, Beijing, 100730 People’s Republic of China

**Keywords:** Endometriosis, Inguinal endometriosis, Hernia, Follow up, Gynecological results

## Abstract

**Background:**

Inguinal endometriosis (IEM) is a rare extra pelvic endometriosis. Here, we study the clinical characteristics, management strategies, and long-term gynecological outcomes of IEM patients at Beijing Chaoyang Hospital.

**Case presentation:**

Three patients presented with a total of four lesions (one on the left side, one on the right side, and one bilaterally). The diameters of the four lesions were 2 cm, 2 cm, 3.5 cm and 1.5 cm, respectively. Two patients were admitted with inguinal hernias. Two patients were admitted with endometrioses—one with ovarian endometriosis and one with pelvic endometriosis. The hernia sac was repaired concomitantly via excision of the round ligament in two patients. One patient underwent a concomitant laparoscopy for gynecologic evaluations, including an ablation to the peritoneal endometriosis, and resection of the left uterosacral ligament endometriosis and pelvic adhesiolysis. All lesions were located on the extraperitoneal portion of the round ligament and were diagnosed histologically. No recurrence was observed in the inguinal region. All patients diagnosed with adenomyosis were treated with medication alone without any complaints.

**Conclusions:**

Inguinal endometriosis can occur simultaneously with pelvic endometriosis. In most cases, a concomitant hernia sac appears together with groin endometriosis. Clinical management should be individualized and performed in tandem with general practitioners and obstetrics & gynecology experts. Pelvic disease, in particular, should be followed-up by a gynecologist.

## Background

As a rare extra pelvic endometriosis, Inguinal endometriosis (IEM) has been reported in 0.3–0.6% of endometriosis patients [1]. IEM is also a possible site of deep endometriosis [2].

As case reports of IEM increase, so does the incidence of IEM [3]. These studies outline the clinical characteristics and the optimal diagnostic and therapeutic strategies for treating IEM [4]. However, long term recovery and gynecological follow-up from IEM patients remains unknown for both gynecologists and general surgeons.

Here, we review cases from 3 patients with IEM who were treated in our hospital. We are the first to report the gynecological results from long-term follow-ups in IEM patients.

## Case presentation

**Fig.1:**
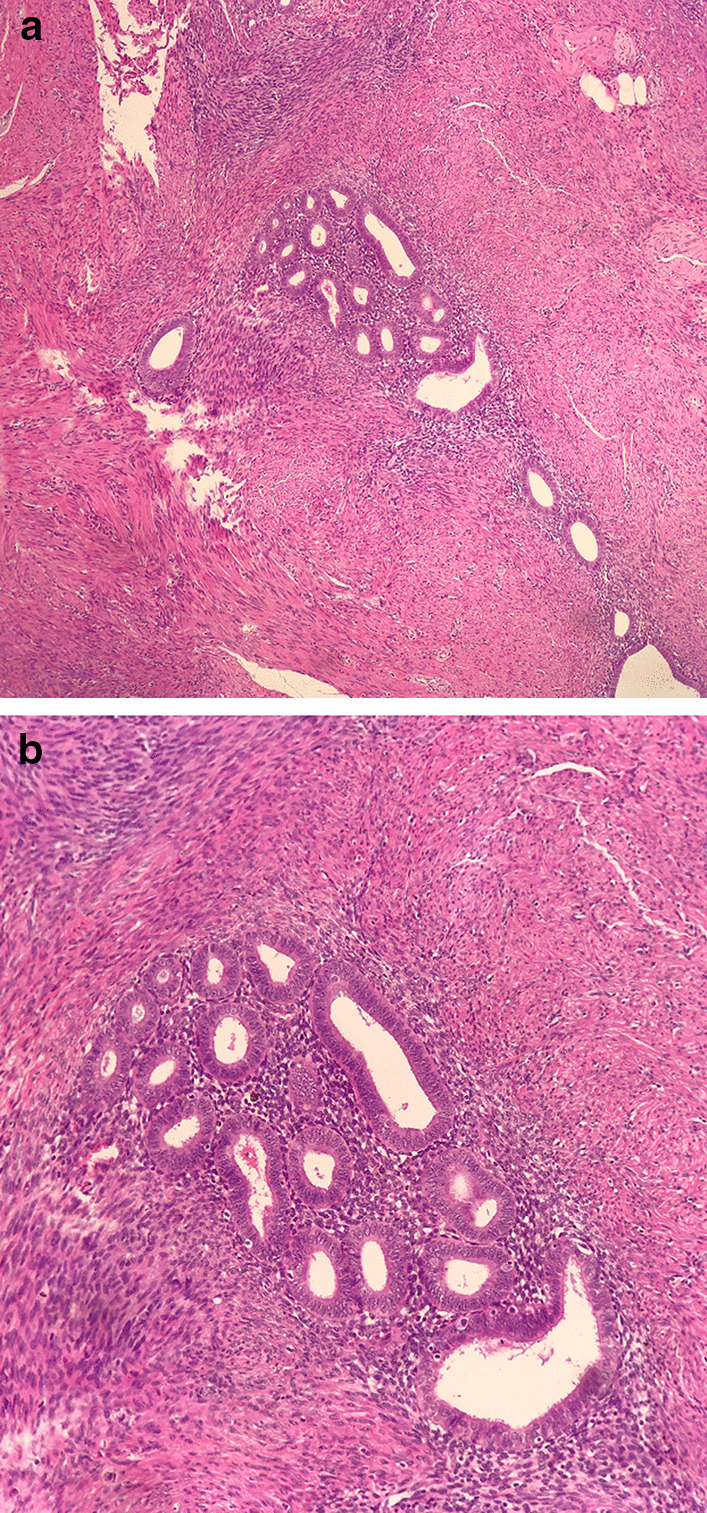
Histopathological examination comprising an endometrial glandular structure lined by columnar epithelium, surrounded by endometrial-type stroma with dense fibrosis; (hematoxylin–eosin, original magnification: **A** ×100; **B**, ×200)

We identified three patients who were admitted to our hospital between 2009 and 2014 with pathologically proven IEM using data from stromal cells within the endometrial glands of the connective tissue in the inguinal lump (Fig. 1A and B). The clinical characteristics of these cases are summarized in Table 1. Institutional review board (IRB) approval was provided.

**Table 1:** Characteristics of three patients with inguinal endometriosis

	Case 1	Case 2	Case 3
Age at diagnosis	32	40	36
Gravidity and parity	2/1	0/0	3/0
Presenting symptoms	Right inguinal mass with catamenial pain for 14mon	Left inguinal incarcerated mass 2y	Left inguinal mass with catamenial pain 6y and right inguinal incarcerated mass 0.5y
History of surgery	Appendectomy in 2004	Right ovarian cystectomy in March, 2011; Infertility	Induced abortion 3 times; dysmenorrhea
Physical findings	Tender 2 cm nodule	Tender 2 cm nodule	Tender 3 cm left inguinal mass, 1.5 cm right inguinal nodule; uterine leiomyoma
MRI findings	isointense and small scattered hyperintense both on T1- and T2-weighted images	NA	Left: hyperintense both on T1- and T2-weighted images Right: hypointense on T1- and T2-weighted images
Type	III	I	Left: III Right: I
Tentative diagnosis	Pelvic and inguinal endometriosis	Incarcerated hernia	Inguinal endometriosis
Operative date	2014-2-14	2012-8-14	2011-11-28
Surgical diagnosis and treatment	Endometriosis of right round ligament excision and pelvic endometriosis ablation	Left round ligament endometriosis excision; repaired the hernia;	Bilateral round ligament endometriosis excision; Hernia sac was found in the right groin and was repaired
Follow-up	70mon Adenomyosis; no recurrence	88mon Adenomyosis; no recurrence	96mon Adenomyosis; no recurrence

**Fig.2:**
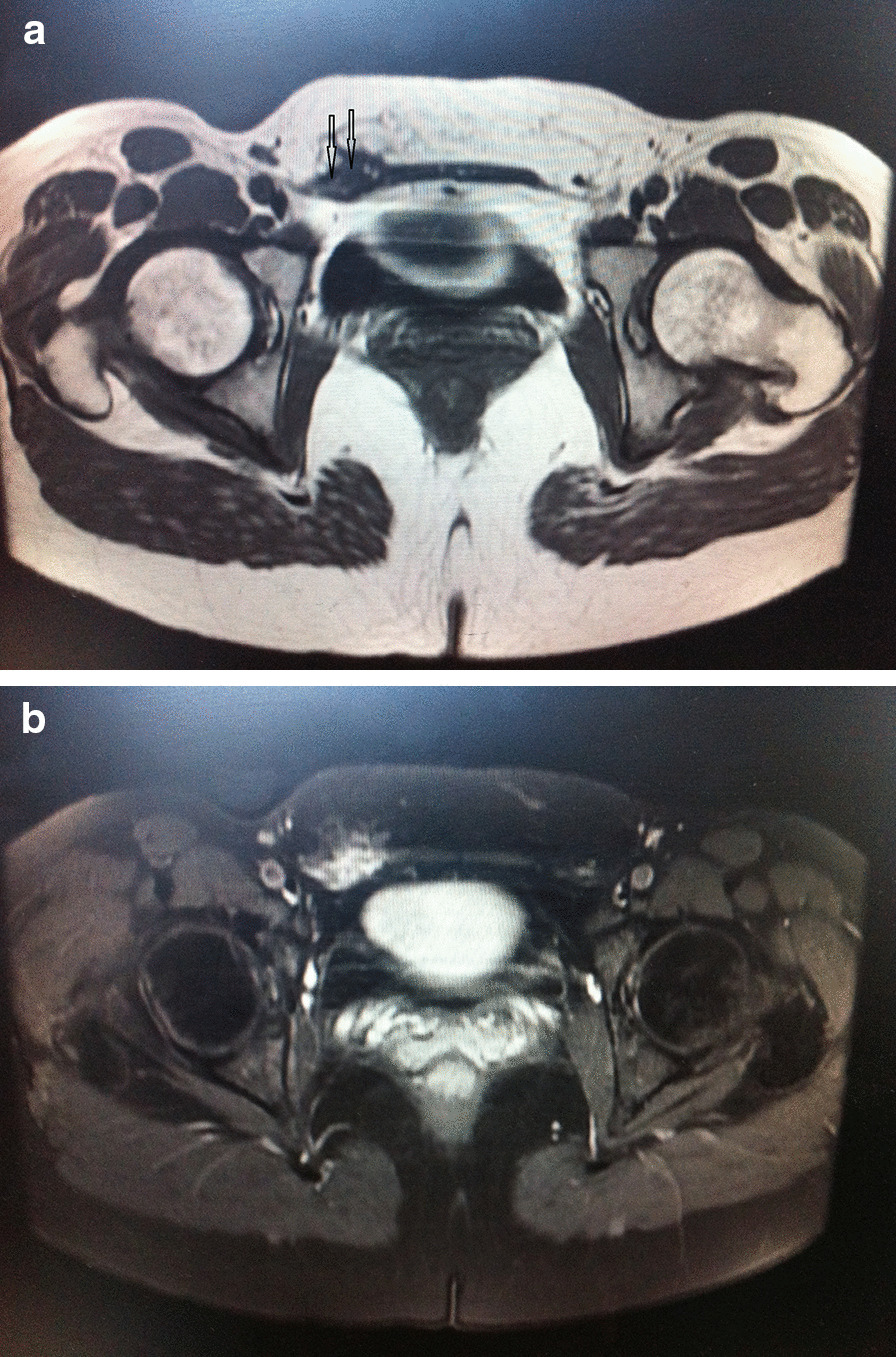
The magnetic resonance imagining reveals an inguinal mass, isointense with muscle, which infiltrate the edge (arrow) of the abdominis rectus muscle, in Axial T1-weighted imagine (**A**). Axial T2-weighted image, obtained at the same level (**B**)

The durations from complaint to diagnosis were 14 months, 6 years, 0.5 years, and 2 years, respectively, for each lesion. Cyclic discomfort in the inguinal region and concomitance with the menstrual period was reported in 2 patients, while 1 complained of dysmenorrhea. Two lesions were reported to change in size during strenuous events such as coughing. One patient previously underwent a right ovarian cystectomy to address endometriosis and infertility 15 months prior. Pre-operative magnetic resonance imaging (MRI) in 2 patients detected a solid, irregular lesion with a hypointense signal and small hemorrhagic foci with hyperintense signals using T1-weighted imaging in the right inguinal area (Fig. 2A, B). 2 patients consulted with a gynecologist and 1 patient was seen by a general surgeon initially. Pre-operative CA125 levels were normal in 1 patient, at 25.6U/ml, and elevated in another, at 78.48 (normal range, 0–35) U/ml. 2 patients were tentatively diagnosed with inguinal (round ligament) endometriosis.

**Fig.3:**
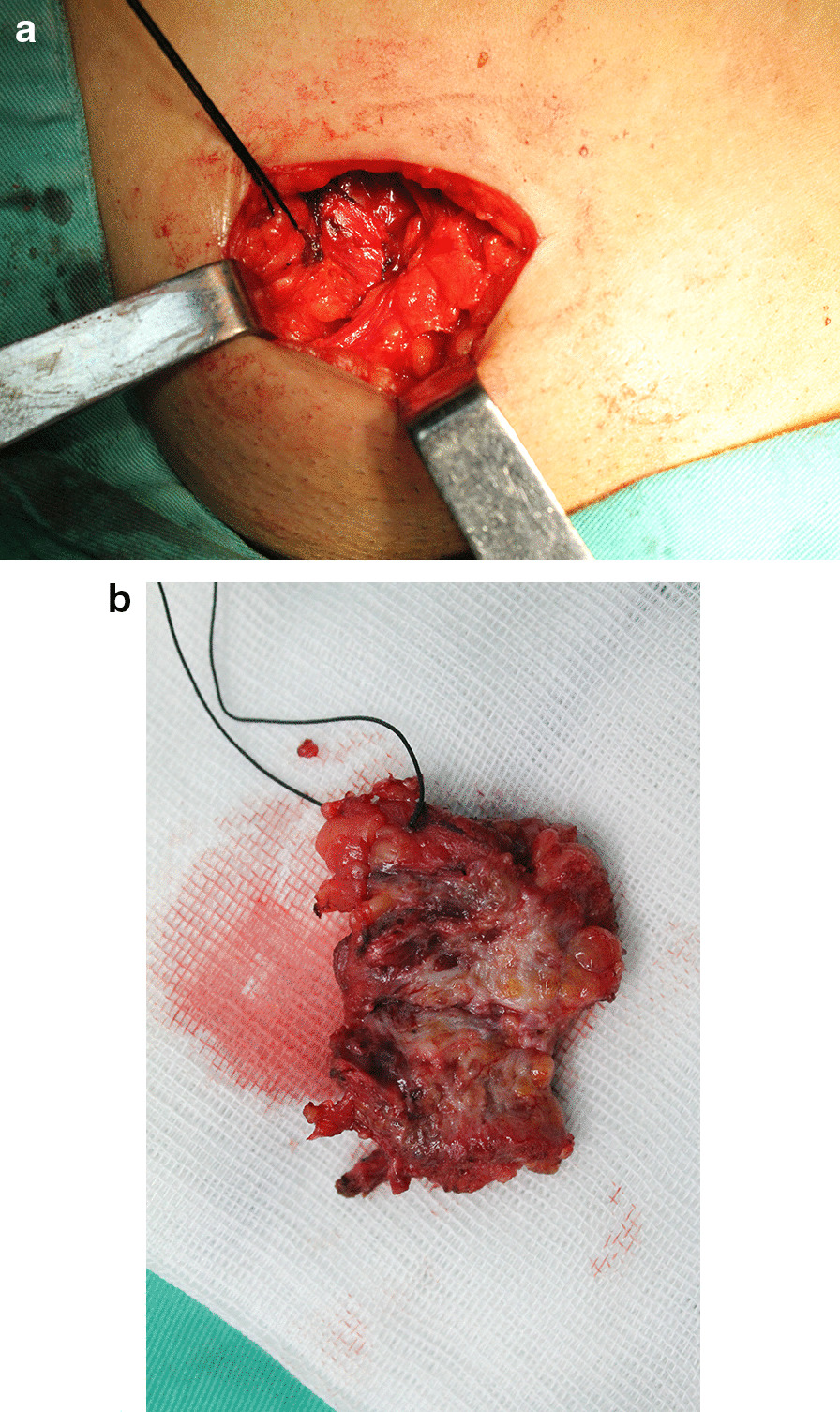
The mass is freed from the adhesions with the internal oblique muscle and the transversalis fascia at the deep inguinal orifice. The inguinal segment of the round ligament was excised with the lesion (**A**). Multi-locular cysts containing dark hemorrhagic content was revealed by gross specimen (**B**)

We assessed 3 patients with a collective total of 4 lesions (1 patient had a lesion on the left side, 1 had a lesion on the right side, and one had bilateral lesions). The diameters of the lesions were 2 cm, 2 cm, 3.5 and 1.5 cm, respectively. 2 patients were diagnosed with inguinal hernias, 2 were diagnosed with endometriosis, one was diagnosed with ovarian endometriosis, and 1 was diagnosed with pelvic endometriosis. Two type III (Case1, left lesion of Case3) lesions adhered to the extraperitoneal portion of the round ligament (Fig. 3A, B).

**Fig.4:**
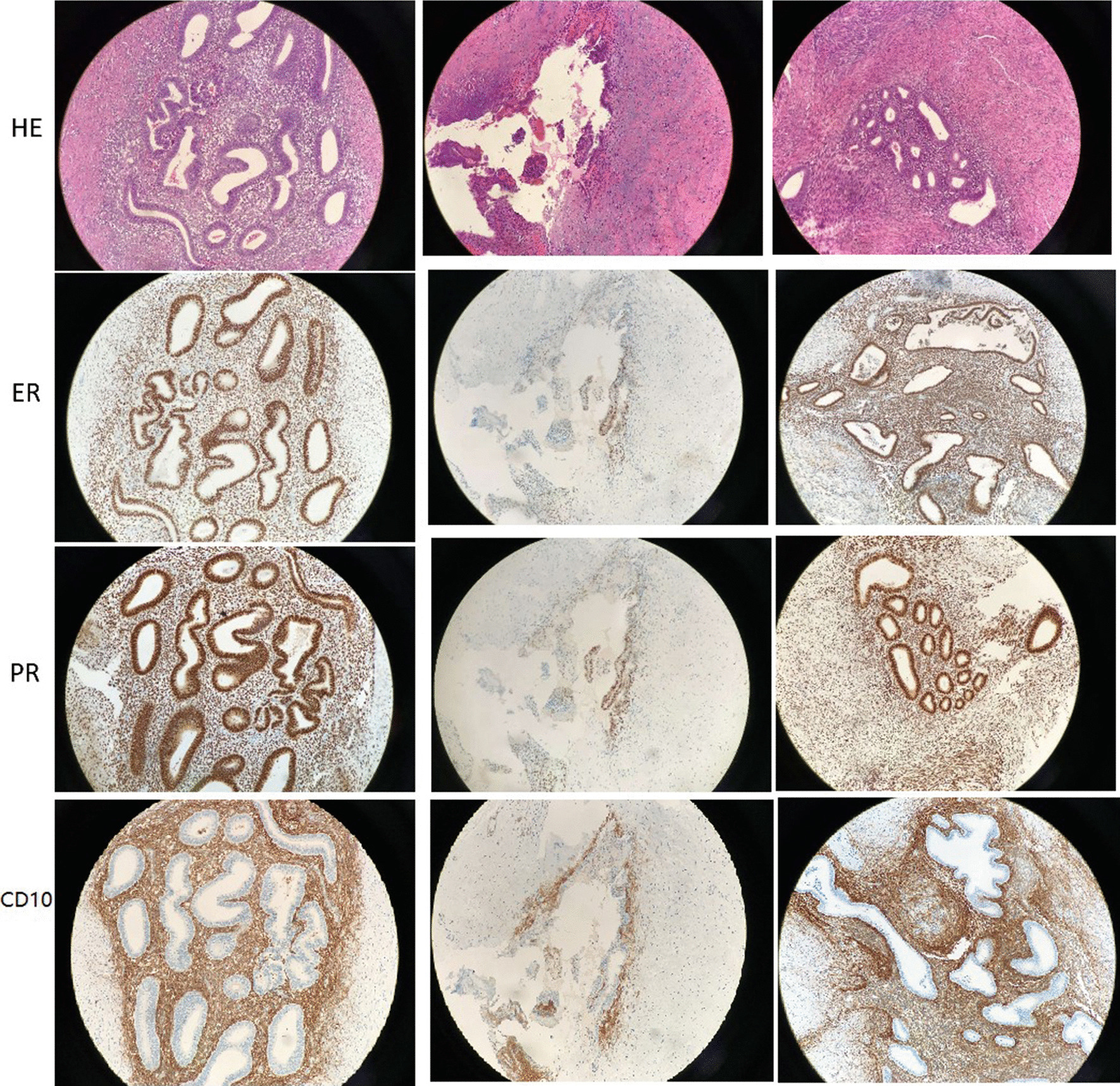
Focal endometriosis, which consisted of endometrial gland and stroma inside the tissue (H&E ×200) of case 1, 2, 3 respectively. ER and PR were positive in glandular and stroma, and CD10 was positive in stroma (× 200)

Two patients underwent procedures to remove the lump and repair the hernia sac. One patient had a laparoscopic ovarian cystectomy in another hospital, and then underwent a procedure 15 months later to remove the lump and repair the hernia. One patient underwent laparoscopy for gynecologic evaluations, including a peritoneal endometriosis ablation, a left uterosacral ligament endometriosis resection and a pelvic adhesiolysis. The patient was discharged two days after the operation. All lesions were diagnosed histologically (Fig. 4) as ER and PR positive in the glandular and stroma (Fig. 4), and CD10 positive in the stroma (Fig. 4). The patients received regular follow-up evaluations and no recurrent lesions were observed. All patients developed adenomyosis, which was treated medicinally and followed-up in our department.

## Discussion

Inguinal endometriosis, a rare form of extra-genital endometriosis, often coincides with pelvic endometriosis. However, a concomitant hernia sac with groin endometriosis should also be considered in the context of inguinal endometriosis. Comprehensive evaluation of patient medical histories should be performed in tandem with imaging and individualized clinical management strategies for IEM patients. Patients who present with both pelvic and inguinal symptoms and are surgical candidates for both procedures should undergo both procedures concomitantly through collaboration between both general surgery and gynecology. Follow-up evaluations should be specifically completed by a gynecologist to check for pelvic disease.

Three clinical types of IEM are reported depending on the site of the lesion: type 1 lesions are located at a hernia sac or hydrocele of Nuck’s canal, type II lesions are on the round ligament, and type III lesions are located under the skin [4]. Type III lesions have been associated with the hernia sac, which is an observation that differs between studies [5, 6]. Two of the 3 patients and 2 of the 4 lesions in our report presented with concomitant hernia sacs in the groin. Inguinal endometriosis often presents concomitantly with hernia sacs in the groin region [7]. Understanding this characteristic could be helpful to effectively direct therapeutic strategies.

Ultrasonography is the first-line diagnostic method for inguinal endometrioses and is used to identify concomitant hernia sacs. However the presentation of inguinal endometriosis in ultrasound is variable, including solid masses, cystic masses, and combined cystic and solid masses [8]. MRI is particularly useful in diagnosing lesions in the extraperitoneal area, and can also be used to identify sub-peritoneal endometriotic deposits [9]. MRI scans of IEM have distinct characteristics [2], including hyperintense T1-weighted images of hemorrhagic micro cysts that provide diagnostic clues for IEM [10].

Differential diagnoses of IEM include inguinal hernia, hydrocele for cystic masses, sarcoma, lymphoma, hematoma, and abscesses for solid masses. Most IEM patients were initially admitted and treated by general surgeons with a false diagnosis of incarcerated hernia. Increased catamenial size and pain during menstruation are hallmarks of an IEM diagnosis. The direct relationship between symptoms and menstruation often successfully rule out other inguinal pathologies [11]. However, surgeons should be aware of the possibility of inguinal endometriosis in fertile women with a lump in the groin region [6].

Surgery involves en bloc radical excision of the lesion along with the extraperitoneal portion of the round ligament [12]. A careful gynecological assessment should be conducted during surgery given that intraperitoneal localization is observed in the majority of cases (91%) [13]. Minimally invasive surgery is the gold standard diagnostic technique for identifying endometriosis [13–16]. Laparoscopy allows for the direct visualization of implants and nodules and aids in excising implants, amplifying minimal lesions, obtaining tissue for diagnosis and stage determination, and treating the disease appropriately.

Hormonal treatment has been underreported as a therapeutic strategy for inguinal endometriosis [14]. It can be an option if the patient does not want to undergo surgery or does not want reoperation after recurrence, and it also could be indicated in patients with concomitant pelvic endometriosis [17]. Arakawa et al. [1] reported that Dienogest effectively managed pain in patients who did not want surgery or reoperation after disease recurrence. The expression of estrogen receptors and progesterone receptors furthers the basis for using hormonal therapies for inguinal endometriosis.

## Conclusions

Long-term follow-up data regarding IEM is limited to a few patients, and operative charts are often missing. Due to its rarity, IEM often lacks thorough investigation. This study provides data from long term follow-ups with IEM patients and provides a deeper understanding of IEM treatment. Follow-up evaluations should continue to be completed by a gynecologist to monitor for intra-abdominal disease and to inform patients of its impact on fertility.

## Data Availability

All data generated or analyzed during this study are included in this published article.

## Abbreviations

IEM: Inguinal endometriosis
MRI: Magnetic resonance imaging
ER: Estrogen receptor
PR: Progesterone receptor
